# *Psoralea glandulosa* as a Potential Source of Anticancer Agents for Melanoma Treatment

**DOI:** 10.3390/ijms16047944

**Published:** 2015-04-09

**Authors:** Alejandro Madrid, Venera Cardile, César González, Ivan Montenegro, Joan Villena, Silvia Caggia, Adriana Graziano, Alessandra Russo

**Affiliations:** 1 Departamento de Química, Facultad de Ciencias Naturales y Exactas, Universidad de Playa Ancha, Avda. Leopoldo Carvallo 270, Playa Ancha, 2340000 Valparaíso, Chile; 2 Department of Biomedical Sciences, University of Catania, V. le A. Doria 6, 95125 Catania, Italy; E-Mails: cardile@unict.it (V.C.); silviacaggia@alice.it (S.C.); acegraziano@tiscali.it (A.G.); 3 Departamento de Química, Universidad Técnica Federico Santa María, Av. España N° 1680, 2340000 Valparaíso, Chile; E-Mail: cesar.gonzalez@usm.cl; 4 Escuela de Obstetricia y Puericultura, Facultad de medicina, Universidad de Valparaíso, Blanco N° 1911, 2340000 Valparaíso, Chile; E-Mail: ivan.montenegro@uv.cl; 5 Centro de Investigaciones Biomédicas (CIB), Escuela de Medicina, Universidad de Valparaíso, Av. Hontaneda N° 2664, 2340000 Valparaíso, Chile; E-Mail: juan.villena@uv.cl; 6 Department of Drug Sciences, Biochemistry Section, University of Catania, V. le A. Doria 6, 95125 Catania, Italy

**Keywords:** *Psoralea glandulosa*, resinous exudates, bakuchiol acetate, A2058 melanoma cells

## Abstract

With the aim of identifying novel agents with antigrowth and pro-apoptotic activity on melanoma cancer, the present study was undertaken to investigate the biological activity of the resinous exudate of aerial parts from *Psoralea glandulosa*, and its active components (bakuchiol (**1**), 3-hydroxy-bakuchiol (**2**) and 12-hydroxy-iso-bakuchiol (**3**)) against melanoma cells (A2058). In addition, the effect in cancer cells of bakuchiol acetate (**4**), a semi-synthetic derivative of bakuchiol, was examined. The results obtained show that the resinous exudate inhibited the growth of cancer cells with IC_50_ value of 10.5 μg/mL after 48 h of treatment, while, for pure compounds, the most active was the semi-synthetic compound **4**. Our data also demonstrate that resin is able to induce apoptotic cell death, which could be related to an overall action of the meroterpenes present. In addition, our data seem to indicate that the apoptosis correlated to the tested products appears, at least in part, to be associated with an increase of reactive oxygen species (ROS) production. In summary, our study provides the first evidence that *P. glandulosa* may be considered a source of useful molecules in the development of analogues with more potent efficacy against melanoma cells*.*

## 1. Introduction

Melanoma is one of the most invasive and deadly forms of skin cancer. Its incidence continues to increase at an alarming rate and, contrary to other neoplasms, a relatively younger population is becoming affected by this tumor. Its treatment mainly depends on the time of diagnosis—if recognized in earlier stages, the surgical excision of this neoplasia is successful; however, late diagnosis leads to an unfavorable fate. Indeed, in the metastatic stage, melanoma becomes very refractory to conventional therapies and nodal metastasis is associated with up to 70% mortality after 10 years [1]. Therefore, research and development of more effective and less toxic drugs by the pharmaceutical industry has become necessary. Many substances present in plants are known to be effective and versatile chemopreventive and antitumoral agents in a number of experimental models of carcinogenesis. In the course of our continuing search for novel agents from natural sources for the treatment and/or prevention of melanoma cancer, we selected the Chilean medicinal plant *Psoralea glandulosa* L. (Fabaceae) for a systematic study on the chemical composition and potential antitumor properties.

**Figure 1:**
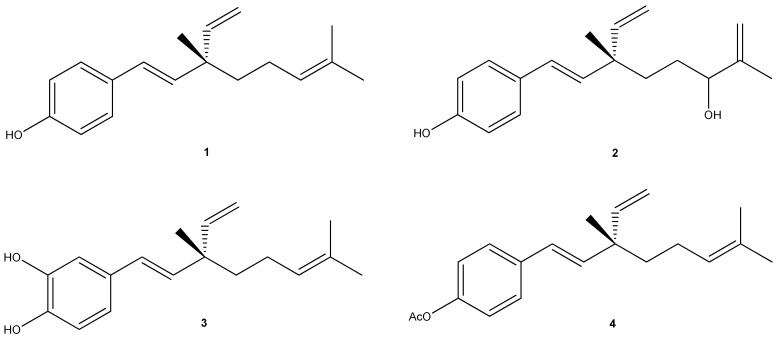
Chemical structures of natural (**1**–**3**) and semi-synthetic (**4**) meroterpenes.

*P. glandulosa* is a resinous shrub, characterized by production of resinous exudates from the glandular trichome that cover the surfaces of its leaves and stems. It is known by the vernacular names “culen” and “hualhua”. *P. glandulosa* has long been used in folk medicine as a vulnerary and for hemorrhoids. It acts as an antiseptic in treatment of infections and skin diseases caused by bacteria and fungus [2,3]. Earlier research on the secondary metabolites of this plant led to the isolation of several compounds: two furanocoumarins, angelicin and psoralen; the drupanin, methyl ester; and the meroterpene, bakuchiol. In addition, the isolation of a mixture of cyclobakuchiols A and B has been reported [4]. Bakuchiol, present in high concentration in resinous exudates [3] and earlier isolated from *Psoralea corylifolia* L., a traditional Chinese medicinal plant used for the treatment of various kinds of disorders, has been shown to exhibit interesting anticancer activities in preclinical studies [5]. Therefore, based on the above rationales and observations, we investigated the biological activity of resinous exudates of the aerial parts of *P. glandulosa*, and its active components bakuchiol (**1**), 12-hydroxy-iso-bakuchiol (**2**) and 3-hydroxy-bakuchiol (**3**) (Figure 1), against human melanoma cancer cells (A2058). To this end, several biochemical parameters were tested, such as cell vitality (MTT assay), cell membrane integrity (lactate dehydrogenase release), genomic DNA fragmentation (COMET assay), and caspase-3 activity. The expression of Bcl-2, Bax, and p53 proteins was also evaluated. Finally, the effect of a semi-synthetic derived from bakuchiol, bakuchiol acetate (**4**) (Figure 1) on cancer cells was examined.

## 2. Results and Discussion

### 2.1. Results

**Figure 2:**
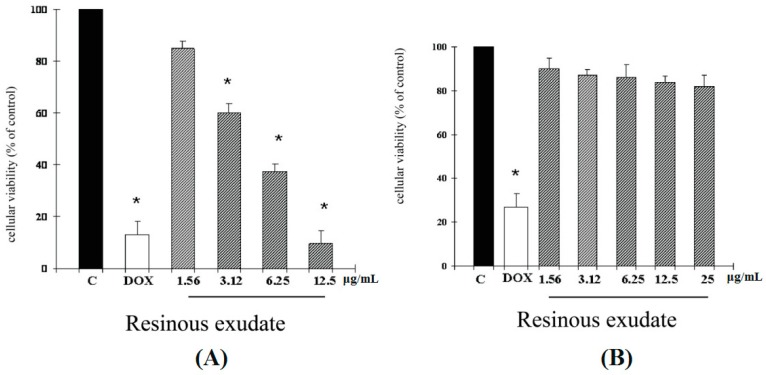
(**A**) Viabilities of A2058 melanoma cells and (**B**) Viabilities of fibroblast cells following treatment with resinous exudate for 48 h were measured as described in “Experimental Section”. Data are expressed as a percentage of the number of viable cells observed with the control, and each column represents the mean ± SD of three experiments, performed in quadruplicate. Significantly different from the control value: *p < 0.001.

Since antiproliferative screening models *in vitro* provide important preliminary data to help select compounds with potential antineoplastic properties for future study, the cytotoxic effects of the resinous exudates on melanoma (A2058) cell lines were evaluated by the MTT assay. The results demonstrated that the resinous exudate exhibited a significant inhibitory effect in A2058 at 48 h of treatment (Figure 2A). Interestingly, the resinous exudate examined, in our experimental conditions, revealed no cytotoxic effect against normal human buccal fibroblast cells (Figure 2B).

Next, we further investigated the cytotoxic effects of pure compounds **1**, **2**, **3**, and **4** on human melanoma cancer cells. The results obtained revealed that all compounds, at non-toxic concentration for normal cells (data not shown), were able to affect the viability of cancer cells (Table 1), but exhibited a lower efficiency than the semi-synthetic derivative compound **4**. Lactate dehydrogenase (LDH) is a soluble enzyme located in the cytosol, which is released into the surrounding culture medium upon cell damage and lysis.

**Table 1:** Cell viability and Lactate dehydrogenase (LDH) release of compounds **1**–**4** tested against A2058 human melanoma cell line.

Sample	IC_50_^a^ (µM)	% LDH ^b^
1	29.3 ± 0.4	5.9 ± 0.6
2	32.3 ± 0.6	6.1 ± 0.5
3	35.1 ± 0.5	6.9 ± 0.4
4	16.9 ± 0.4	7.3 ± 0.4
Doxorubicin	0.20 ± 0.01	-
Negative Control	100	6.5 ± 0.9

^a^ IC_50_: value represents the concentration that inhibited cell vitality by 50%. Each value represents the mean ± SD of three experiments, performed in quadruplicate; ^b^ % LDH: value expressed as percentage of LDH released into the cell medium with respect to total LDH, in A2058 cells untreated and treated for 48 h with compounds **1**–**4** at 40 µM concentration. Negative control: Cultures received DMSO alone.

LDH activity in the culture medium can, therefore, be used as an indicator of membrane integrity, and thus a measurement of cytotoxicity [6]. No statistically significant increase in LDH release was observed in A2058 cells treated with the resinous exudate at 3.12–25 μg/mL concentrations (Table 2). Again, no statistically significant increase in LDH release was observed in melanoma cancer cells treated with the pure compounds at 40 µM concentration (Table 1). Impaired apoptosis signaling is common in cancer cells and may play an important role in tumor initiation and progression. Resistance of cancer cells to apoptosis is especially deleterious because it not only enhances the spontaneous growth of tumors but also makes them resistant to host defense mechanisms as well as various forms of therapy [7].

**Table 2:** Lactate dehydrogenase (LDH) release in A2058 cells treated with different concentrations of resinous exudate of *P. glandulosa*.

Sample	Treatment	%LDH ^a^
Resinous exudate	3.12 μg/mL	7.5 ± 0.6
	6.25 μg/mL	9.7 ± 0.5
	12.5 μg/mL	8.9 ± 0.7
	25 μg/mL	8.8 ± 0.6
H_2_O_2_	1 μM	5.8± 0.7
	1000 μM	71.3± 1.7 *
Negative control	0.25%	7.5 ± 0.7

^a^ The values are the mean ± SD of three experiments performed in quadruplicate; * Significant *vs.* control untreated cells (*p* < 0.001) Stock solution of resinous exudate was prepared in DMSO and the final concentration of this solvent was kept constant at 0.25%. Negative control: Cultures received DMSO alone.

**Figure 3:**
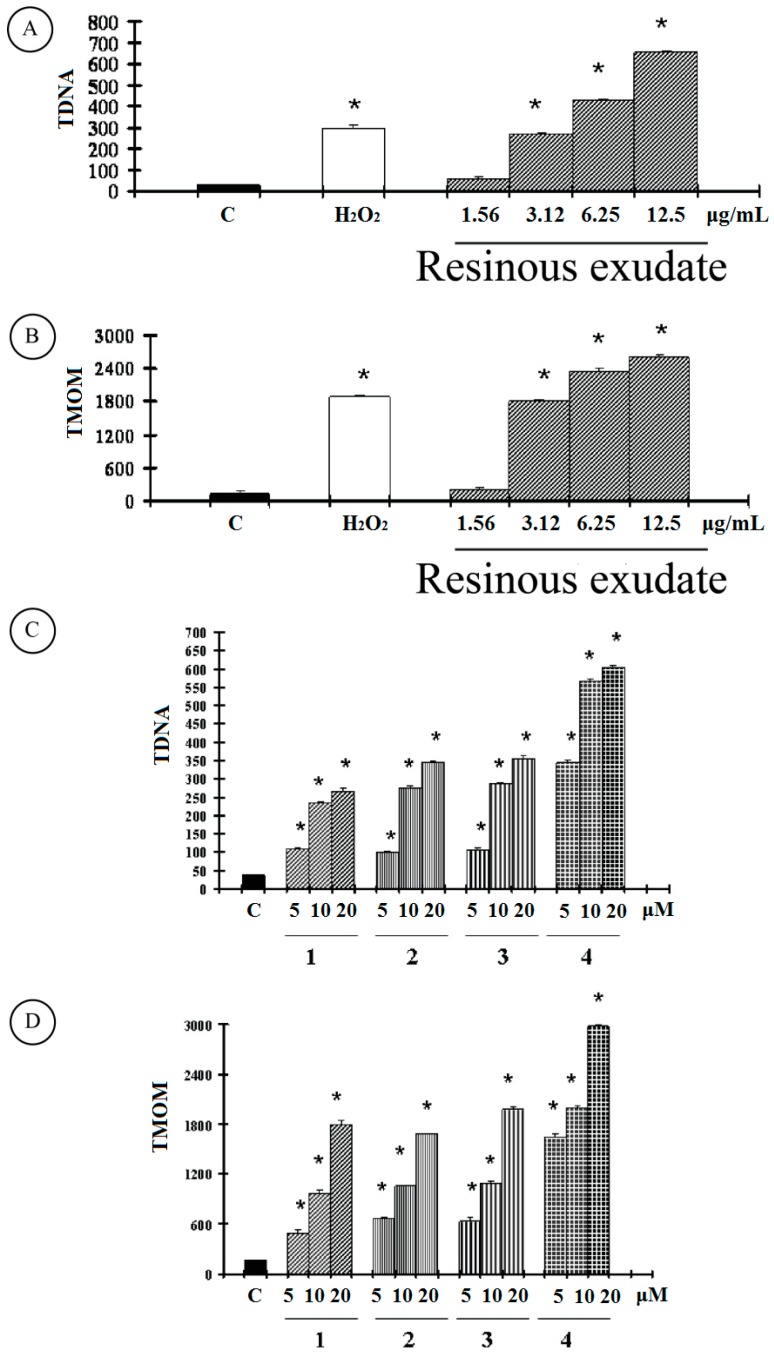
Comet assay in A2058 cancer cells untreated and treated at different concentrations for 48 h with (**A**,**B**) resinous exudates; with (**C**,**D**) compounds **1**–**4** were measured as described in “Materials and Methods”. The values are the mean ± SD of three experiments performed in quadruplicate. Significantly different from the control value: *p < 0.001.

Therefore, we next addressed whether the cytotoxicity induced by the resinous exudate is linked to apoptosis. Nuclear DNA was analyzed using single-cell gel electrophoresis (SCGE), known as COMET assay, and a sensitive method for the visualization of DNA damage measured at the level of individual cells. The COMET assay also allowed us to distinguish apoptotic from necrotic cells based on the DNA fragmentation pattern [8]. The COMET pattern significantly differs between apoptotic and control cultures, as well as between apoptotic and necrotic cultures. Quantification of the COMET data, in our experimental condition, is reported as TDNA and TMOM in (Figure 3).

As reported for the positive control, hydrogen peroxide (1 μM), an apoptotic inductor in cancer cell lines, clearly shows evidence of an increase in both TDNA and TMOM after treatment with the resinous exudate in A2058 cells. These findings on genomic DNA damage seem to suggest that this natural product triggers apoptotic cell death, since data in the literature indicates that only COMETs with high values of TMOM and TD can be related to apoptosis [9]. Active caspases cleave to several important intracellular proteins, leading to the morphological and biochemical changes associated with apoptosis, such as oligonucleosomal fragmentation of chromosomal DNA. Caspase-3 is the major executioner caspase in the caspase cascade, so subsequent experiments were performed to characterize the role of activation of this protein in cell growth inhibition mediated by tested resinous exudate.

As shown in Table 3, the activity of caspase-3, measured by p-nitroaniline (pNA), released from the specific caspase substrate, and reported as OD405 nm/mg protein, was significantly increased in A2058 cells treated with the resinous exudate and hydrogen peroxide (1 μM). The treatment of melanoma cancer cells with the pure compounds bakuchiol, 12-hydroxy-iso-bakuchiol, 3-hydroxy-bakuchiol, and bakuchiol acetate also induced high values of TMOM (Figure 3D), which are correlated with an increase in caspase-3 activity (Table 3).

**Table 3:** Caspase-3 activity in A2058 cancer cells untreated and treated with resinous exudate and compounds **1**–**4** at different concentrations for 48 h.

Sample	Treatment	OD_405_ nm/mg Protein
Resinous exudate	3.12 μg/mL	0.41 ± 0.03 *
	6.25 μg/mL	0.55 ± 0.06 *
	12.5 μg/mL	0.84 ± 0.05 *
Compound **1**	5 μM	0.45 ± 0.05 *
	10 μM	0.63 ± 0.04 *
	20 μM	0.89 ± 0.09 *
Compound **2**	5 μM	0.38 ± 0.06 *
	10 μM	0.55 ± 0.04 *
	20 μM	0.77 ± 0.09 *
Compound **3**	5 μM	0.39 ± 0.04 *
	10 μM	0.62 ± 0.04 *
	20 μM	0.81 ± 0.06 *
Compound **4**	5 μM	0.65 ± 0.03 *
	10 μM	0.73 ± 0.04 *
	20 μM	0.97 ± 0.06 *
H_2_O_2_	1 μM	0.89 ± 0.09 *
Negative control	0.25%	0.27 ± 0.06

The values are the mean ± SD of three experiments performed in quadruplicate. * Significant *vs.* control untreated cells (*p* < 0.001). Hydrogen peroxide (1 μM) was used as a positive control. Stock solution of extract was prepared in DMSO and the final concentration of this solvent was kept constant at 0.25%.

**Figure 4:**
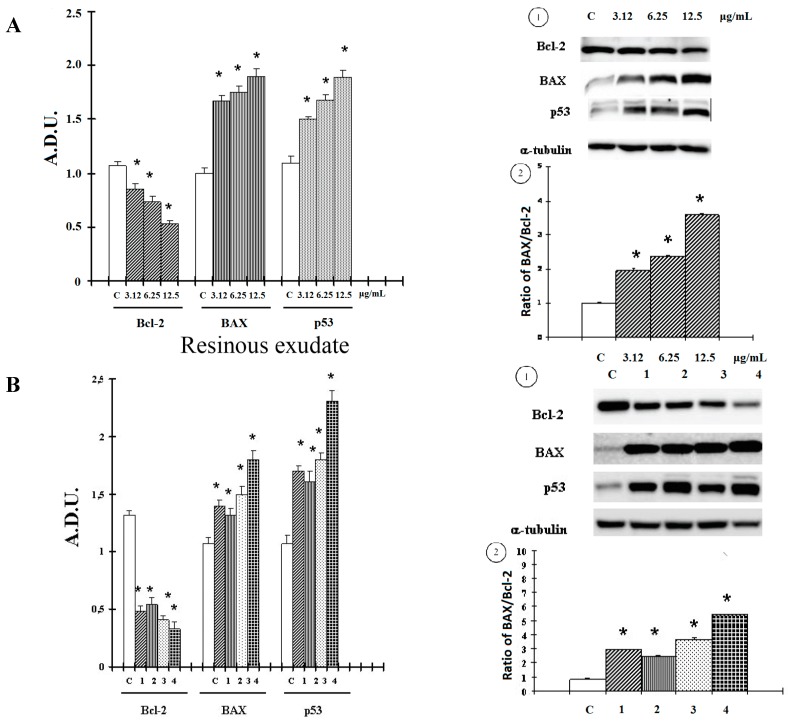
Levels of Bcl-2, Bax and p53 proteins in A2058 cells untreated and for 48 h were measured as described in “Materials and Methods” treated with (**A1**) resinous exudate and (**B1**) compounds **1**–**4**; Bax/Bcl2 ratio treated with (**A2**) resinous exudates and (**B2**) compounds **1**–**4**. Values are expressed as arbitrary densitometric units (A.D.U.) corresponding to signal intensity present on the autoradiography of western blots. Representative blots are reported above each graph. Each value represents the mean ± SD of three experiments, performed in quadruplicate. * Significant *vs.* control untreated cells (*p* < 0.001).

However, synthetic compound **4** exhibited a major effect. Bcl-2 family proteins are key players in survival and apoptosis, and the Bcl-2 protein expression in tumors has prognostic power [10]. The mechanisms by which the Bcl-2 family proteins regulate apoptosis are different. Ultimately, they govern decisive steps that determine whether certain caspase family cell death proteases remain quiescent or become active [11]. In our experimental conditions, we observed that treatment with resinous exudate (Figure 4A), for active components present (**1**–**3**) (Figure 4B), down-regulated anti-apoptotic Bcl-2 protein expression in melanoma cells. We next evaluated the effect of treatment on pro-apoptotic Bax. As shown in Figure 4A1,B1, an increase in Bax protein, in conjunction with the more pronounced decrease in Bcl-2, for the resinous exudate and meroterpene compounds, at 3.12–12.5 μg/mL and 20 μM, respectively, shifted the Bax/Bcl-2 ratio in favor of apoptosis (Figure 4A2,B2). The p53 family plays a central role in apoptosis, acting as stress sensors of the cell and triggering the activation of various pro-apoptotic genes [11]. Therefore, Western blotting was done to check the expression of p53 in response to the resinous exudate and pure meroterpenes. As shown in Figure 4A,B, a p53 up-regulation was detected in our experimental conditions.

**Figure 5:**
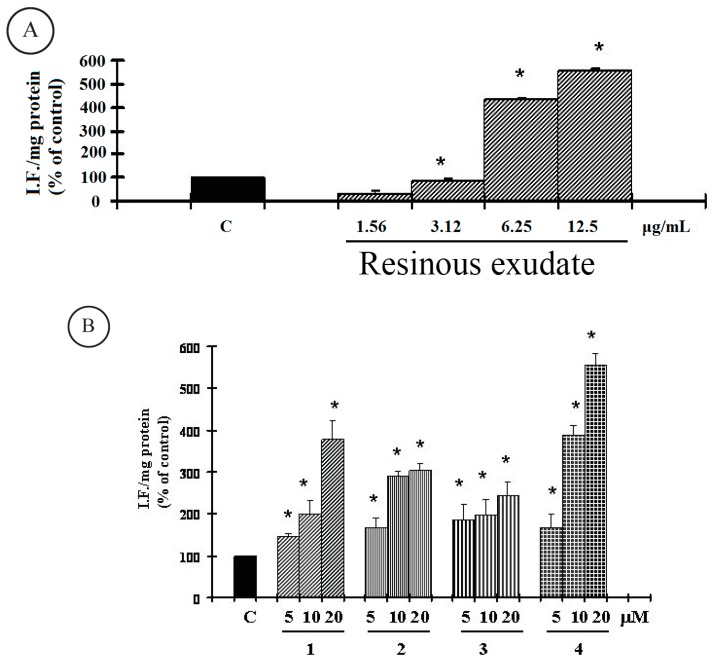
Reactive oxygen species (ROS) determination were measured as described in “Experimental Section”, in A2058 cells untreated and treated for 48 h with resinous exudate (**A**) and pure compounds **1**–**4** (**B**) at different concentrations. The values are the mean ± SD of three experiments performed in quadruplicate. * Significant *vs.* control untreated cells (*p* < 0.001).

ROS has been reported to be involved in cell death induced by a variety of stimuli and different antitumoral agents. We therefore examined whether tested resinous-exudate-induced cell death may be correlated to an elevation of ROS. To assess changes in intracellular ROS levels, we employed a DCFH-DA oxidation-sensitive fluorescent probe. DCFH-DA can be taken up into cells, and then oxidized by ROS to its fluorescent derivative DCF. We found that the DCF fluorescence increased in a concentration-dependent manner in A2058 cells treated with resinous exudate and meroterpene compounds (Figure 5A,B).

### 2.2. Discussion

Use of herbal remedies in the treatment of various diseases, including skin diseases, has a long tradition around the world. Furthermore, herbal medicine is gaining increasing attention in the study of skin diseases [12,13]. In the present study, we investigated the effect of resinous exudate from the aerial parts of *P. glandulosa* and its active components (compounds **1**–**3**, and semi-synthetic derivate bakuchiol acetate (**4**)) on melanoma. *P. glandulosa* has been used as a tonic or as a useful antiseptic in treating infections and skin diseases caused by bacteria and fungus. The resinous exudates exerts antifungal [2] and antioxidative [3] activities. Bakuchiol exerts chemopreventive [14], antimicrobial [15], and anti-inflammatory [16] activities. Bakuchiol also induces apoptosis in rat liver myofibroblasts through bax mitochondrial translocation and caspase 3 activation [17]. Compound **2** exerts anti-proliferative effect on cultured A549 cell lung, and lower effects on SK-OV-3 (ovary) and SK-MEL-2 (melanoma) human cells [18]; compound **3** exerts cytotoxic effects in TA3/Ha mammary carcinoma cells as a result of the inhibition of mitochondrial electron transport, leading to apoptosis of treated cells [19].

Previous studies show that bakuchiol exhibits a slightly higher inhibitory activity on the proliferation of SK-MEL-2 melanoma human cells [18] and inhibitory effects on viability and melanin production in B16 mouse melanoma cells [20] *in vitro*. Our results, according to literature data, demonstrate that the resinous exudates was able to inhibit the growth of cancer cells with IC_50_ value at 10.5 μg/mL, which is most due to the synergistic effect provided by the meroterpenes **1**–**3**, evidenced in our experimental conditions, and as previously suggested [21].

However, the more potent efficiency of bakuchiol acetate suggests that the level of cytotoxicity is also related to the nature and position of the substituent in the aryl moiety, and could be related, at least in part, to the alkyl group. As an aside, it has been reported that the introduction of electron-donating groups in the bakuchiol molecule improves cytotoxicity [22]. However, the most interesting finding of this study was the observation that the semi-synthetic derived compound **4** exhibited a major cytotoxic effect against cancer cells (especially melanoma). The isolated meroterpenes from *P. glandulosa* cytotoxic activity increases with the growth of the lipophilicity on the terpenic molecule, a trend which is also apparent with other derivates’ esters of bakuchiol, against human tumor cell lines like lung, ovarian, liver, mama or another human melanoma cell line, for instance [5,18].

Apoptosis is a major cellular homeostatic mechanism in normal skin and plays an important role in the defense against damaged or transformed cells. In cutaneous cells, there is a homeostatic relationship between cell proliferation and apoptosis. Alterations in either cell proliferation or cell death can lead to a loss of growth control, and thus play a major role in the process of tumorigenesis. Defects of apoptotic pathways influence drug resistance as well, and because of these defects, chemotherapy often fails. Studies have suggested that the resistance of human melanoma to apoptosis is an important mechanism underlying this cancer’s aggressiveness and its poor response to chemotherapeutic agents [7]. The induction of apoptosis in tumor cells is considered very useful in the management and therapy of cancer, including melanoma [7]. Synthesis or modification of known drugs continues as an important aspect of research.

There is a continued need for new templates to be used in the design of potential chemotherapeutic agents. Significantly, natural products are providing such templates. Recent studies on tumor inhibitory compounds of plant origin have yielded an impressive array of novel structures [23]. It is thus considered important to screen apoptotic inducers from plants, either in the form of crude extracts or as components isolated from them. Bakuchiol is known to be inducers of cytotoxicity by triggering apoptosis [22]; it is able to reduce mitochondrial membrane potential (ΔΨm), to induce caspase 9/3 activation, p53 and Bax up-regulation, as well as Bcl-2 down-regulation [22]. Consistent with this approach, and according to previous studies, our data suggest that the resinous exudate from the aerial parts of *P. glandulosa*, such as the active meroterpenes, is able to trigger apoptotic death in melanoma cancer cells.

In fact, a high DNA fragmentation (Comet assay), not correlated to LDH release (which is a marker of membrane breakdown), occurred in melanoma cells treated with both this natural product and pure compounds **1**–**4**. The hypothesis of apoptosis induction in our experimental conditions was reinforced by a significant (*p* < 0.001) increase of the caspase-3 enzyme activity.

Investigations have demonstrated that reactive oxygen species (ROS) acts as a mediator of apoptosis [24]. The increase of reactive oxygen species and the loss of mitochondrial membrane potential (ΔΨm) were typical phenomena in the process of apoptosis [25]. Thus, anticancer agents that act by targeting mitochondria and causing reactive oxygen species generation to promote cancer cell death have been in development [26]. Reactive oxygen species trigger the apoptotic cascade [27]. Generation of reactive oxygen species results in a decrease of ΔΨm, as well as in the elevation of Bax, and reduction of, Bcl-2 levels. Subsequently, the release of cytochrome c and activation of caspases leads to apoptotic changes [27]. Our results, according to these literature data, suggest an involvement of ROS production in the pro-apoptotic capacity of the examined extract and pure compounds. In fact, compounds **1**–**4** were able to increase significantly, and in a concentration-dependent manner, ROS generation. Of interest, it has been demonstrated that bakuchiols, such as the phenolic compounds epigallocatechingallate, quercetin, gallic acid, curcumin and eugenol, act not only as antioxidants, but also as prooxidants [22].

## 3. Experimental Section

### 3.1. Plant Material

The aerial parts of *P. glandulosa* were collected in Lo Orozco, Valparaíso province (V Región), Chile, in November 2013 and identified by Forest Engineer Patricio Novoa from “Jardín Botánico Nacional”, Viña del Mar, Chile.

### 3.2. Chemical and Reagents

Dichloromethane (CH_2_Cl_2_) for the resin extraction of the aerial parts of *P. glandulosa* was purchased from Sigma Chemical Co. (St. Louis, MO, USA). Column chromatography (CC) was performed on silica gel (200–300 mesh, Merck, Santiago, Chile) for the isolation of compounds. Compounds **1**–**3** were monitored by TLC (Merck, Santiago, Chile), and spots were visualized by 25% H_2_SO_4_-H_2_O reagent. Dimethyl sulfoxide (DMSO), Hydrogen peroxide solution (30% (*w*/*w*) in H_2_O), doxorubicin (99% pure) was purchased from Sigma Chemical Co. (St. Louis, MO, USA). Other reagents and solvents were purchased from commercial suppliers and used were analytical grade.

### 3.3. Preparation of the Resinous Exudates

The resinous exudate of *P. glandulosa* (30 g, 3%) was obtained by dipping fresh plant material (1 kg) in cold CH_2_Cl_2_ for 25–30 s. at room temperature and the filtered solution was then concentrated under reduced pressure [3].

### 3.4. Isolation of Natural Compounds **1**–**3**

Compounds **1**–**3** were isolated from resinous exudate of *P. glandulosa* L. (Fabaceae). The isolation methodology of pure compounds was performed according to reported procedures [3,28]. Compounds **1**–**3** were identified by optical rotation, and spectroscopic data, including ^1^H- and ^13^C-NMR and comparisons with data reported in the literature [2,3,4,28]. The % purity of compounds **1**–**3** (bakuchiol (95%), 3-hydroxy-bakuchiol (93%) and 12-hydroxy-iso-bakuchiol (95%)) were confirmed by analytical HPLC.

### 3.5. Procedure for Synthesis of Compound **4**

The compound **4** prepared from a solution of bakuchiol (1.0 g, 4 mmol) in DCM (25 mL) by the conventional method [29], to give acetate bakuchiol **4** (1.17 g, 98,6%) as semisolid.

### 3.6. Cell Culture

A human melanoma cell line, A2058, was obtained from American Type Culture Collection (Rockville, MD, USA). The cells were maintained in DMEM, containing 10% fetal calf serum, 2.0 mM l-glutamine, 100 U/mL penicillin, 100 μg/mL streptomycin, and 2 mM non-essential amino acids. Normal human non-immortalized buccal fibroblast cells were grown in Dulbecco’s modified Eagle’s medium (DMEM) supplemented with 10% fetal calf serum (FCS), 100 U/mL penicillin, 100 μg/mL streptomycin, and 25 μg/mL fungizone. The cells were plated at a constant density to obtain identical experimental conditions in the different tests, and thus achieve high measurement accuracy.

In the MTT assay, the cancer cells were plated at 6 × 10^3^ cells per well in a 96-well, flat-bottomed, 200 μL microplate, and at 2 × 10^4^ cells per well for normal human non-immortalized buccal fibroblast cells in a 96-well, flat-bottomed, 200 μL microplate. In other tests, the cells were plated at 8 × 10^5^ cells (2 mL) per 35 mm culture dish. After 24 h incubation at 37 °C under a humidified 5% carbon dioxide to allow cell attachment, the cells were treated with different concentrations of the resinous exudate and pure compounds (**1**–**4**), and incubated for 48 h under the same conditions. This treatment time was chosen since no effect of the resinous exudate and pure compounds examined has ever been observed before 48 h of treatment, at least for the parameters examined by us. Stock solution of the resinous exudate and pure compounds was prepared in dimethylsulfoxide (DMSO) and the final concentration of this solvent was kept constant at 0.25%. Control cells received DMSO alone.

### 3.7. MTT Bioassay

Cell viability was performed as described previously [30]. Briefly, cells were incubated at 37 °C in a humidified 5% CO_2_/95% air mixture and treated with the resinous exudate and pure compounds at different concentrations for 48 h. Four hours before the end of the treatment time, 20 μL of 0.5% 3(4,5-dimethyl-thiazol-2-yl)2,5-diphenyl-tetrazolium bromide (MTT) in phosphate buffer saline (PBS) were added to each microwell. Cells were washed once before adding MTT. After four hours of incubation at 37 °C, the supernatant was removed and replaced with 100 μL of DMSO. The optical density of each well sample was measured with a microplate spectrophotometer reader (Digital and Analog Systems, Rome, Italy) at 550 nm. Doxorubicin was used as positive control.

### 3.8. Lactate Dehydrogenase (LDH) Release

The Lactate dehydrogenase (LDH) activity was spectrophotometrically measured in the culture medium and in the cellular lysates at 340 nm by analyzing NADH reduction during the pyruvate-lactate transformation, as previously reported [30]. Briefly, cells were lysed with 50 mM Tris-HCl + 20 mM EDTA pH 7.4 + 0.5% sodium dodecyl sulfate (SDS), disrupted by sonication, and centrifuged at 13,000× *g* for 15 min. The assay mixture (1 mL final volume) for the enzymatic analysis contained: 33 μL of sample (5–10 μg of proteins) in 48 mM PBS pH 7.5, plus 1 mM pyruvate and 0.2 mM NADH. The percentage of LDH released was calculated as percentage of the total amount, considered as the sum of the enzymatic activity present in the cellular lysate and of that in the culture medium. A Hitachi U-2000 spectrophotometer (Hitachi, Tokyo, Japan) was used. Hydrogen peroxide solution, an apoptotic inductor in cancer cell lines, was used as standard.

### 3.9. Activity of Caspase-3

The activity of caspase-3 was determined by using the Caspase colorimetric assay Kit Sigma-RBI (St. Louis, MO, USA). This assay measures the cleavage of a specific colorimetric caspase substrate, acetyl-Asp-Glu-Val-Asp p-nitroanilide (Ac-DEVD-pNA). pNA (p-nitroaniline) is released from the substrate upon cleavage by caspase. Free pNA produces a yellow color that is monitored by a Hitachi U-2000 spectrophotometer (Hitachi) at 405 nm. The caspase-3 activity was measured in cell lysates. The cell pellets were incubated at 4 °C for 20 min with lysis buffer containing 50 mM HEPES (1-piperazineethane sulfonic acid, 4-(2-hydroxyethyl)-monosodium salt), pH 7.4, 5 mM CHAPS (3[(3-cholamidopropyl)dimethylammonio]-propanesulfonic acid), and 5 mM DTT (1,4dithio-dl-threitol). The lysed cells were centrifuged at 16,000× *g* for 15 min at 4 °C, and the supernatants were analyzed immediately according to the analysis procedure described in the manufacture’s protocol. The total protein content was determined as previously described [31]. Each sample was evaluated, and the results are reported as OD405 nm/mg protein and compared to relative control. Hydrogen peroxide solution, an apoptotic inductor in cancer cell lines, was used as standard.

### 3.10. DNA Analysis by COMET Assay

The presence of DNA damage was examined as previously described [32] by single cell gel electrophoresis (COMET assay). Briefly, 0.8–1 × 10^5^ cells were mixed with 75 μL of 0.5% low melting agarose (LMA) and spotted on slides. The “minigels” were maintained in lysis solution (1% *N*-laurosil-sarcosine, 2.5 M NaCl, 100 mM Na2EDTA, 1% Triton X-100, 10% DMSO, pH 10) for 1 h at 4 °C, and then denatured in a high pH buffer (300 mM NaOH, 1 mM Na2EDTA, pH 13) for 20 min, and finally electrophoresed in the same buffer at 18 V for 45 min. At the end of the run, the “minigels” were neutralized in 0.4 M Tris-HCl, pH 7.5, stained with 100 μL of ethidium bromide (2 μg/mL) for 10 min, and scored using a fluorescence microscope (Leica, Wetzlar, Germany) interfaced with a computer. Software (Leica-QWIN) allowed assessment of the quantitative and qualitative extent of DNA damage by measuring: (a) tail length (TL), intensity (TI) and area (TA); (b) head length (HL), intensity (HI) and area (HA). Finally, the program using these parameters calculated the level of DNA damage as: (i) the percentage of the fragmented DNA (TDNA); and (ii) tail moment (TMOM). The tail moment is defined as the product of the percentage of DNA in the tail of the comet and TD value, which is obtained by calculating the distance between the center of mass of the comet head and the center of mass of the tail. The percentage of DNA in the comet tail was calculated as the rate of the fluorescence intensity in the comet tail relative to the total fluorescence; 100 randomly selected cells were analyzed per sample. Hydrogen peroxide solution, an apoptotic inductor in cancer cell lines, was used as standard.

### 3.11. Western Blot Analysis

The expression of Bcl-2, Bax, and p53 proteins was evaluated by Western blot analysis. Briefly, the untreated and treated cell line A2058 was washed twice with ice-cold PBS and collected with a lysing buffer (10 mM Tris-HCl plus 10 mM KCl, 2 mM MgCl_2_, 0.6 mM PMSF, and 1% SDS, pH 7.4). After cooling for 30 min at 0 °C, the cells were sonicated. Twenty micrograms of total protein, present in the supernatant, were loaded on each lane and separated by 4%–12% Novex Bis-Tris gel electrophoresis (NuPAGE, Invitrogen, Milan, Italy). Proteins were then transferred to nitrocellulose membranes (Invitrogen) in a wet system. The transfer of proteins was verified by staining the nitrocellulose membranes with Ponceau S and the NovexBis-Tris gel with Brillant blue R. Membranes were blocked in Tris buffered saline containing 0.01% Tween-20 (TBST) and 5% non-fat dry milk at 4 °C overnight. Bcl-2 (SAB2500154, Sigma Aldrich, Milan, Italy) (1:500 dilution), Bax (B3428, Sigma-Aldrich) (1:2000 dilution), rabbit polyclonal anti-p53 (FL-393; sc-6243, Santa Cruz Biotechnology, Santa Cruz, CA, USA) (1:300 dilution) and α-tubulin (T5326; Sigma-Aldrich) (1:5000 dilution) antibodies were diluted in TBST and membranes incubated for 2 h at room temperature. Antibodies were detected with horseradish peroxidase-conjugated secondary antibody using the enhanced chemiluminescence detection Supersignal West Pico Chemiluminescent Substrate (Pierce Chemical Co., Rockford, IL, USA). Bands were measured densitometrically with ImageJ software and their relative density calculated based on the density of the α-tubulin bands in each sample. Values were expressed as arbitrary densitometric units corresponding to signal intensity.

### 3.12. Reactive Oxygen Species Assay

Reactive oxygen species (ROS) determination was performed by using a fluorescent probe 2',7'-dichlorofluorescein diacetate (DCFH-DA), as previously described [30]. DCFH-DA diffuses through the cell membrane, and is enzymatically hydrolysed by intracellular esterases as well as oxidized to the fluorescent 2',7'-dichlorofluorescein (DCF) in the presence of ROS. The intensity of fluorescence is proportional to the levels of intracellular oxidant species. One hundred microliters of 100 μM DCFH-DA, dissolved in 100% methanol, was added to the cellular medium where the acetate group is not hydrolyzed, and the cells were incubated at 37 °C for 30 min. After incubation, cells were lysated and centrifuged at 10,000× *g* for 10 min. The fluorescence (corresponding to the radical species-oxidized 2',7'-dichlorofluorescein, DCF) was monitored spectrofluorometrically using a Hitachi F-2000 spectrofluorimeter (Hitachi): excitation 488 nm, emission 525 nm. The total protein content was determined as previously described [31]. Each sample was evaluated, and the results are reported as fluorescence intensity/mg protein and compared to relative control.

### 3.13. Statistical Analysis

Each value represents the mean ± SD of three experiments performed in quadruplicate, for which the mean and standard deviation for each value was calculated. Results were analyzed using one-way ANOVA followed by Dunnett’s post-hoc test for multiple comparisons with control. All statistical analyses were performed using the statistical software package SYSTAT, version 9 (Systat Inc., Evanston, IL, USA). Differences were considered significant at *p* < 0.05.

## 4. Conclusions

Our results confirm the effectiveness of the resinous exudates of *P. glandulosa* in reducing cell viability, in A2058 melanoma cells and in agreement with previous investigations, which provides *in vitro* scientific support for the use of several Fabaceae species for cancer-related diseases. In this context, the resinous exudates spark scientific interest from a chemical point of view due to the presence of bioactive metabolites; as an alternative solution to current pathologies, and, from a commercial point of view, due to their fast processing and the low investment required. The results obtained show that the resinous exudate inhibited the growth of cancer cells after 48 h of treatment, while, for pure compounds, the most active was the semi-synthetic compound **4**. In addition, we show the first *in vitro* evidence that *P. glandulosa* may be considered a source of molecules useful for the development of analogues with more efficacy in potential treatments *in vivo* against melanoma cells.
